# Exploring Therapeutic Potential of Catalase: Strategies in Disease Prevention and Management

**DOI:** 10.3390/biom14060697

**Published:** 2024-06-14

**Authors:** Shehwaz Anwar, Faris Alrumaihi, Tarique Sarwar, Ali Yousif Babiker, Amjad Ali Khan, Sitrarasu Vijaya Prabhu, Arshad Husain Rahmani

**Affiliations:** 1 Department of Medical Laboratory Technology, Mohan Institute of Nursing and Paramedical Sciences, Mohan Group of Institutions, Bareilly 243302, India; shehwazanvar25@mipharmacy.org.in; 2 Department of Medical Laboratories, College of Applied Medical Sciences, Qassim University, Buraydah 51452, Saudi Arabia; 3 Department of Basic Health Sciences, College of Applied Medical Sciences, Qassim University, Buraydah 51452, Saudi Arabia; 4 Department of Biotechnology, Microbiology and Bioinformatics, National College (Autonomous), Tiruchirapalli 620001, India; vijaybio0624@gmail.com

**Keywords:** oxidative stress, antioxidant, catalase, therapeutic potential, limitations

## Abstract

The antioxidant defense mechanisms play a critical role in mitigating the deleterious effects of reactive oxygen species (ROS). Catalase stands out as a paramount enzymatic antioxidant. It efficiently catalyzes the decomposition of hydrogen peroxide (H_2_O_2_) into water and oxygen, a potentially harmful byproduct of cellular metabolism. This reaction detoxifies H_2_O_2_ and prevents oxidative damage. Catalase has been extensively studied as a therapeutic antioxidant. Its applications range from direct supplementation in conditions characterized by oxidative stress to gene therapy approaches to enhance endogenous catalase activity. The enzyme’s stability, bioavailability, and the specificity of its delivery to target tissues are significant hurdles. Furthermore, studies employing conventional catalase formulations often face issues related to enzyme purity, activity, and longevity in the biological milieu. Addressing these challenges necessitates rigorous scientific inquiry and well-designed clinical trials. Such trials must be underpinned by sound experimental designs, incorporating advanced catalase formulations or novel delivery systems that can overcome existing limitations. Enhancing catalase’s stability, specificity, and longevity in vivo could unlock its full therapeutic potential. It is necessary to understand the role of catalase in disease-specific contexts, paving the way for precision antioxidant therapy that could significantly impact the treatment of diseases associated with oxidative stress.

## 1. Introduction

The equilibrium of the cellular redox environment is maintained by the production of reactive oxygen species (ROS) and reactive nitrogen species (RNS) and the removal of these species by antioxidant enzymes and small-molecular-weight antioxidants. A redox cycle controls oxidative metabolic processes and cell cycle activities. It does this by preventing the production of free radicals by eliminating oxidizing molecules enzymatically, interrupting free radical chain reactions, and fixing damaged molecules back into their proper structures to keep cells from becoming more vulnerable to oxidative stress in the future [1].

Moreover, ROS can act as second messengers in the regulation of cell growth and differentiation due to the intricate intracellular interaction between oxidizing and reducing equivalents. Low-level local ROS are significant because they regulate critical transcription factors (NFκB/IκB, Nrf2/KEAP1, AP-1, p53, HIF-1) and act as redox-signaling molecules in a variety of pathways involved in ensuring the maintenance of cellular homeostasis (MAPK/ERK, PTK/PTP, PI3K-AKT-mTOR). As a result, ROS have the ability to influence several cellular processes, such as apoptosis, migration, differentiation, and proliferation [2].

ROS can work by directly interacting with certain receptors or by redox-activating signaling pathway components including transcription factors, protein phosphatases, and kinases. Additionally, ROS collaborate with intracellular Ca2+ in signaling pathways that control the ratio of cell division to cell cycle arrest and cell death [3].

**Figure 1:**
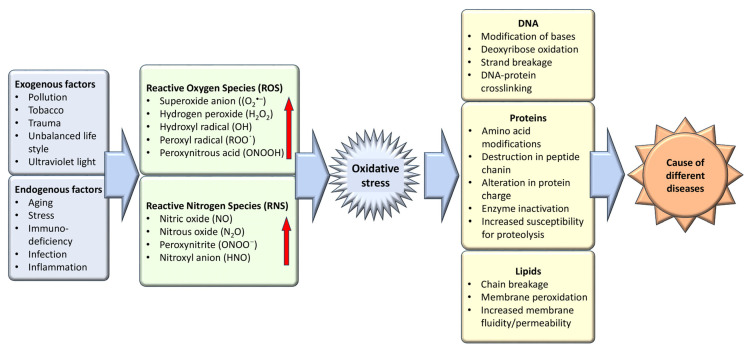
Exogenous and endogenous factors are responsible for the generation of oxidative stress which is the causative agent of different diseases.

Oxidative stress, an imbalance favoring oxidants over antioxidants, disrupts redox signaling and causes molecular damage [4]. Maintaining a steady-state redox balance within the open metabolic system is vital, as it generates a basal redox tone at a particular setpoint. Deviations from this balance trigger a stress response, highlighting oxidative stress as a critical component of physiological redox signaling and control [5]. Molecular damage caused by free radicals to proteins, lipids, and nucleic acids—vital structural components of cellular membranes and nuclei—underscores the importance of maintaining a balance between antioxidants and free radicals for health preservation (Figure 1). Therefore, the effective management of oxidative stress is key in preventing and treating a wide range of diseases, including diabetes, cancer, atherosclerosis, coronary artery diseases, inflammatory and liver disorders, cardiovascular conditions, cataracts, nephrotoxicity, and neurodegenerative diseases related to aging [6]. The body leverages enzymatic and non-enzymatic antioxidants sourced from the diet or synthesized endogenously to preserve redox homeostasis. Given the adverse effects associated with the long-term use of synthetic antioxidants—such as skin allergies, gastrointestinal disorders, and an elevated cancer risk due to contaminants, harmful byproducts, and toxic solvents—there is an increasing shift towards investigating safe, natural antioxidants [7].

**Figure 2:**
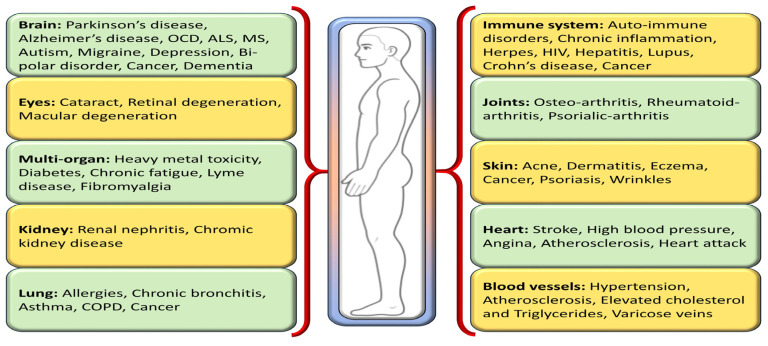
Different oxidative stress-mediated diseases in various parts of the human body.

One way to disentangle the relationship between oxidative stress and various diseases is by the addition or administration of exogenous or endogenous redox-active substances, which have the ability to either restore or disturb the cells’ current redox status. Antioxidants, a class of naturally occurring compounds found in food, mitigate physiological oxidative stress. Given the body’s continuous radical generation due to oxygen utilization, antioxidants are crucial in preventing and repairing cellular damage linked to diverse health issues, including cancer, diabetes, macular degeneration, and heart disease [8] (Figure 2). As efficient free radical scavengers, antioxidants preclude or repair cellular damage by delaying or preventing other molecules from undergoing oxidation [9].

Living organisms have an endogenous antioxidant defense system that is categorized into enzymatic and non-enzymatic antioxidants, each harboring unique mechanisms, action sites, and effects defined by their diverse compositions. This diversity underscores the respective role each antioxidant plays in the body. Among them, the antioxidant enzyme network, encompassing glutathione reductase (GRd), catalase (CAT), superoxide dismutase enzymes (SODs), and glutathione peroxidase (GPx), forms an effective defense system [10]. SOD is a primary antioxidant defense, offering significant therapeutic potential [11,12]. Ceruloplasmin, ferritin, transferrin, and albumin are the non-enzymatic components of preventative antioxidants that participate in the first line of defense in blood plasma. These proteins bind transition metal ions (such as iron and copper) to prevent the synthesis of new reactive species [13].

The endogenous antioxidant system is frequently insufficient in neutralizing ROS during mitochondrial respiration and during normal cellular metabolism using molecular oxygen such as NADPH oxidase and xanthine oxidase. Therefore, exogenous antioxidants such as vitamin C and VE, carotenoids, and polyphenols are needed to balance our oxidant/antioxidant system. Exogenous antioxidants are helpful in guarding against free radicals and are crucial in preventing a variety of diseases. However, a high concentration of these exogenous antioxidants might lead to chronic disorders like cancer. Thus, whereas exogenous antioxidants at low doses—which are preventative doses—reduce oxidative stress, at high doses—which are therapeutic doses—they can increase the selective killing of cancer cells and the efficacy of standard therapy by boosting ROS generation with a pro-oxidative effect [14]. Exogenous as well as endogenous antioxidants can cooperate to preserve or restore redox equilibrium. Interacting ROS and antioxidants are regarded as functionally linked redox-active chemicals; they are essential parts of an organism’s redox processes [15].

Hydrogen peroxide is considered a widespread chemical and it can be absorbed through our diet, exhaled, and/or excreted [16]. In mammals, H_2_O_2_ (H_2_O_2_) is produced through enzymatic as well as non-enzymatic processes. The mitochondria, NADPH oxidases, and other oxidases (free or membrane-bound) are examples of endogenous H_2_O_2_ sources. Under the action of three superoxide dismutases, the superoxide anion radical is transformed into H_2_O_2_. Essentially, the non-enzymatic process originates from the reduction of the •O_2_^−^ anion by e^−^ and H^+^. This reduction of O_2_ occurs in the cellular mitochondrial matrix throughout the mitochondrial respiration pathway (complexes I, II, III, and IV). ATP-synthase-introduced protons are utilized in the reduction of molecular O_2_ to •O_2_^−^, H_2_O_2_, and ultimately H_2_O. Superoxide dismutases, which are found in the mitochondria, cytosol, or extracellular space, quickly convert the partial reduction of oxygen that occurs during aerobic respiration or by multiple oxidases into H_2_O_2_ [17].

In physiological oxidative stress, H_2_O_2_ functions as a key redox signaling component [17]. It is commonly accepted that H_2_O_2_ is a cytotoxic agent, and that antioxidant defense enzymes must function to reduce its concentration. In actuality, H_2_O_2_ is not very reactive when transition metal ions are not present. There may be more exposure to H_2_O_2_ in some human tissues than is often believed [16].

H_2_O_2_ can act as a messenger to transport a redox signal from the point of its formation to a target site due to its physicochemical characteristics. Of all the oxygen metabolites, H_2_O_2_ is thought to be the most appropriate for redox signaling. One way to regulate redox is by transcriptional regulation or by controlling a specific enzyme activity. A variety of transcription factors are affected by hydrogen peroxide: mammalian cells (AP-1, NRF2, CREB, HSF1, HIF-1, TP53, NF-κB, NOTCH, SP1, and SCREB-1), bacteria (OxyR and PerR), and lower eukaryotes (Yap1, Maf1, Hsf1, and Msn2/4) [18]. H_2_O_2_ can be neutralized by a variety of enzymes. Among these enzymes are peroxidases such cytochrome c peroxidase and NADH peroxidase, as well as catalase and glutathione peroxidase [19].

Catalase (CAT) plays a vital role in cellular defense mechanisms by mitigating oxidative stress caused by reactive oxygen species (ROS). It exhibits one of the highest catalytic efficiencies among enzymes, with a single catalase molecule converting millions of H_2_O_2_ molecules into water and oxygen every second. This remarkable turnover rate underscores the enzyme’s critical function in protecting cells from oxidative damage [20]. Catalase’s high Km makes it most effective at breaking down high amounts of hydrogen peroxide, which can be found in peroxisomes, the subcellular organelle that contains the majority of catalase. Since CAT has a very rigid, stable structure, it is comparatively more resistant to changes in temperature, pH, and proteolysis, making it a unique antioxidant enzyme. Catalases are unique in that they do not require an analogous cellular reducing agent. Since the peroxisome is the center of H_2_O_2_ synthesis because of fatty acid β-oxidation, photorespiration, oxidative stress, and purine catabolism, they are mostly present there [21]. Catalase excels at breaking down large levels of hydrogen peroxide. Its high Km is the reason for this. By means of a mechanism known as radical scavenging, the small-molecule antioxidants eliminate the ROS and remove them. In this category, carotenoids, glutathione (GSH), vitamin C, and vitamin E are the primary antioxidants. Enzymes (SOD, CAT, and GSHPx) and sacrificial proteins (albumin) are examples of large-molecule antioxidants that absorb ROS and stop them from damaging other vital proteins [22]. The catalytic values of catalase make it unique compared to small-molecule antioxidants.

The conserved gene structures and motif patterns of catalase genes are strikingly comparable among species, and they are also similar across species that belong to the same evolutionary branch. These findings imply that catalase genes have been conserved throughout evolution and provide compelling evidence for evolutionary taxonomic identity [23]. Comparative genomic evaluations of the three main families of antioxidant enzymes—CAT, PRX, and GPX—were conducted in 19 species with high-quality genomes spanning 10 metazoan phyla, ranging from chordates to sponges. The antioxidant enzyme families CAT and PRX are both ancient in evolution and have a high degree of conservation, as the research shows [24].

## 2. Structural Characterization of Catalase

**Figure 3:**
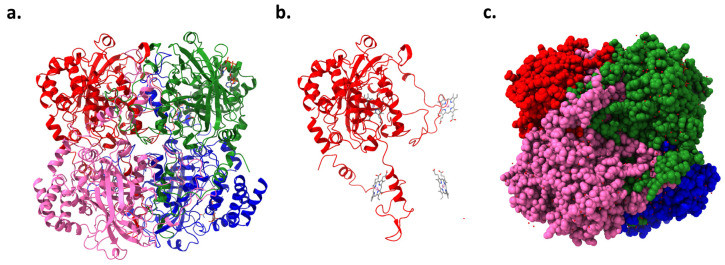
Human erythrocyte catalase (PDB code: 1DGB): (**a**) ribbon representation showing four catalase chains, (**b**) one chain of catalase and position of iron protoporphyrin IX prosthetic groups in the other three associated chains, and (**c**) space-filling model of catalase.

The CAT gene, located on chromosome 11 in humans, encodes the enzyme catalase. This enzyme has been a subject of scientific study for over a century, with numerous isolations, purifications, and characterizations performed on catalase from various organisms [25]. High-resolution crystallography has resolved the structures of 16 monofunctional catalases, revealing that each is a tetramer (Figure 3a,c). The four active sites within these tetramers include a pentacoordinate iron protoporphyrin IX prosthetic group (Figure 3b) complexed with a tyrosine [26]. Additionally, some catalases incorporate a tightly bound NADPH cofactor at the subunit periphery, enhancing the enzyme’s stability and regulatory capacity. Catalase’s tetrameric structure features four polypeptide chains, each with over 500 amino acids, which facilitate the decomposition of hydrogen peroxide into water and oxygen [27]. The iron-rich heme groups within each subunit’s active site are central to its activity, deeply embedded, and vital for interacting with the substrate. Structurally, catalase exhibits a highly conserved architecture across different species, including a central β-barrel core, stabilizing the enzyme through evolutionary adaptations [26].

Each subunit incorporates an amino-terminal arm, an antiparallel eight-stranded β-barrel domain, a wrapping domain, and an α-helical domain. These components are crucial for the enzyme’s functionality, from substrate interaction in the β-barrel domain to structural interconnections facilitated by the wrapping loop. Predominantly located within peroxisomes, human catalase features a monofunctional, heme-containing design essential for interacting with hydrogen peroxide through ferric porphyrin IX. The structural complexity is enhanced by the quartet of domains within each subunit, notably the distal histidine in the N-terminal arm critical for catalytic action. The α-helical domain’s role extends to NADPH binding, highlighting these domains’ multifaceted regulatory and activity-related functions [28,29]. Contrasts between different catalase types, such as those from animal erythrocytes and Proteus mirabilis, uncover structural variabilities that affect substrate specificity and enzymatic activity, demonstrating the adaptability and diversity within this enzyme family [30,31]. The intricate arrangement of these domains underscores the enzyme’s stability and functional specificity, pivotal for its critical role in cellular antioxidant defenses.

## 3. Functions of Catalase

Emerging Catalase (EC 1.11.1.6) is a pivotal enzyme in the defense against oxidative stress. It is renowned for rapidly decomposing hydrogen peroxide into water and molecular oxygen, processes essential for maintaining cellular integrity amidst oxidative challenges. This peroxisomal, heme-based enzyme demonstrates high substrate specificity and turnover rates, enabling it to respond to significant fluctuations in reactive oxygen species (ROS) levels within cells [32,33]. Its action mitigates potential damage from byproducts like hydroxyl radicals and peroxynitrite, which can accrue under oxidative stress [34]. The role of hydrogen peroxide extends beyond a mere reactive molecule; it functions as a signaling molecule involved in various physiological processes such as immune response activation, cell proliferation, and apoptosis. However, hydrogen peroxide can be detrimental when in excess, stressing the importance of tightly regulated ROS levels for optimal cellular function [34].

The structural framework of catalase, characterized by its homotetrameric formation and the presence of ferriprotoporphyrin IX at its active sites, underpins its catalytic efficiency and versatility. These heme groups enable not only the decomposition of hydrogen peroxide but also the breakdown of other substrates like nitric oxide and peroxynitrite, showcasing the enzyme’s broad functional scope [35,36]. Catalase’s global ubiquity across aerobic life underscores its essential role in antioxidant defense mechanisms, reinforcing its indispensability in maintaining cellular redox equilibrium and protecting against oxidative damage [31]. Also, catalase exhibits peroxidative activity, oxidizing substances such as ethanol and phenolic compounds, especially when low hydrogen peroxide concentrations are present, which points to its adaptability and broader metabolic significance [37,38].

The enzyme’s mechanism involves the initial interaction with hydrogen peroxide to form a high-valent iron intermediate known as Compound I, which features an oxyferryl heme and a p-cationic porphyrin protein radical. This intermediate is key to the catalase catalytic cycle, where it can further react with hydrogen peroxide, returning the iron ion to its ferric state while releasing water and oxygen [20,39]. A deepening understanding of catalase also extends into clinical and therapeutic realms. For instance, the study by Eiro et al. (2022) delves into how catalase deficiency impacts the viability of cryopreserved mesenchymal stromal cells, demonstrating its importance in potential regenerative medicine applications [40]. Furthermore, the explorations into catalase–peroxidase KatG in Escherichia coli provide insights into how these enzymes can differentiate their functionalities to exhibit both catalytic and peroxidative actions, expanding our understanding of their roles in cellular biochemistry [41,42]. Understanding catalase’s multifaceted roles not only furthers basic biological knowledge but also aids in refining strategies for coping with oxidative stress in various health states, thereby highlighting its critical role in bioscience and medicine [43].

## 4. Mechanism of Action of Catalase

**Figure 4:**
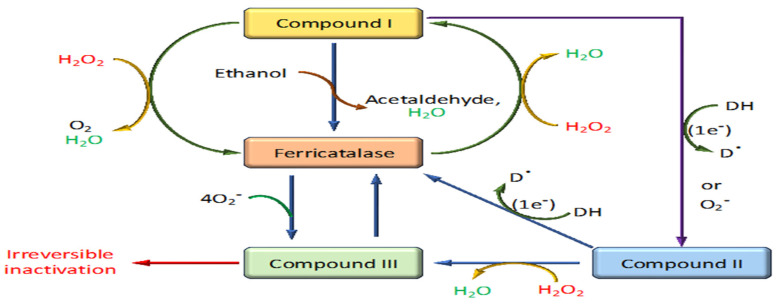
Reaction mechanism of catalase through different stages.

The mechanism of action of catalase is an efficient two-step process crucial for protecting cells from oxidative damage by rapidly decomposing hydrogen peroxide into water and oxygen [44] (Figure 4). This highly efficient catalytic process involves distinct phases centered around the enzyme’s active site, which contains a heme group equipped with an iron ion [39].

### 4.1. Binding of Hydrogen Peroxide

The initial step involves binding a hydrogen peroxide molecule to the ferric (+3) iron atom in the heme group at the catalase’s active site. This interaction prepares the enzyme for the oxidative phase of the reaction [44,45].

### 4.2. Formation of Compound I (Cpd I)

Upon hydrogen peroxide binding, the enzyme undergoes a two-electron oxidation, reducing the bound hydrogen peroxide to water. This process transforms the ferric iron into a high-valent iron intermediate known as Compound I. Compound I features a configuration with an iron-oxo species (Fe=O) and a porphyrin radical cation, preparing it for further reaction [46].

### 4.3. Reaction with a Second Hydrogen Peroxide Molecule

This highly reactive intermediate can interact with a second hydrogen peroxide molecule. During this interaction, the reaction cleaves the O-O bond of hydrogen peroxide, leading to the release of molecular oxygen and additional water molecules. This step effectively restores the iron to its ferric state [47].

### 4.4. Release of Products and Resetting of the Enzyme

After the catalytic cycle, water and oxygen, the decomposition products, are released from the enzyme’s active site. The enzyme is then reset and ready to process another hydrogen peroxide molecule [44]. The reduction of Compound I back to its resting state can occur via the transfer of a hydrogen atom, involving either a His-mediated (Fita–Rossmann) mechanism where a distal histidine acts as an acid–base catalyst or a direct mechanism that allows for a hydrogen atom transfer. Each pathway involves two one-electron transfers, highlighting the nuanced role of distal residues in facilitating these reactions and emphasizing the atomic and electronic reorganizations crucial to the catalase mechanistic pathway [47]. Overall, this mechanism exemplifies catalase’s role as one of the most efficient enzymes in the antioxidant defense system of aerobic organisms, facilitating the conversion of millions of hydrogen peroxide molecules each second, thereby protecting cells from the potential damage of oxidative stress.

## 5. Role of Catalase in Different Diseases

Under conditions of heightened oxidative stress, such as those observed in aging, inflammation, and various diseases, catalase’s expression and enzymatic functionality become pivotal in averting cellular injury and preserving cellular integrity. The role of catalase in mitigating oxidative stress underscores its utility as a therapeutic avenue in a spectrum of pathological states characterized by increased reactive oxygen species (ROS) production (Figure 1 and Figure 2). Delving into the sophisticated modalities by which catalase modulates oxidative stress responses is fundamental for formulating precise therapeutic strategies to bolster cellular resilience and avert diseases attributed to oxidative damage [31]. A myriad of health conditions, including diabetes mellitus, vitiligo, cardiovascular disorders, Wilson disease, hypertension, anemia, specific dermatological ailments, Alzheimer’s disease, bipolar disorder, and schizophrenia, have been associated with a deficiency or malfunctioning of catalase. Additionally, acatalasemia, a rare genetic anomaly characterized by impaired catalase activity (also recognized as Takahara disease), emerges due to aberrations in catalase function [31]. The linkage between altered catalase expression and an array of disorders has been substantiated by findings indicating specific mutations in the catalase gene correlating with diminished catalase activity, with such associations being documented in the context of diabetes, hypertension, vitiligo, Alzheimer’s disease, and acatalasemia [48,49]. This evidence highlights the critical involvement of catalase activity regulation in the pathophysiology of a diverse set of diseases, illuminating the scope for targeting catalase function in therapeutic interventions aimed at mitigating oxidative stress-induced cellular and molecular derangements.

### 5.1. Catalase and Neurodegenerative Diseases

Alzheimer’s disease (AD) is characterized by the accumulation of amyloid-β (Aβ), neurofibrillary tangles, cognitive decline, mitochondrial dysfunction, and oxidative stress-induced damage. In AD, where oxidative stress plays a significant role in neuronal damage, researchers have scrutinized the status of catalase activity in AD pathology. Studies have noted variations in catalase levels and activity within the brains of individuals with Alzheimer’s disease, suggesting a potential correlation between catalase dysfunction and neurodegeneration progression [50,51] (Figure 2).

Moreover, investigations have explored how catalase impacts beta-amyloid accumulation, a hallmark feature of AD pathology. The enzyme’s role in modulating oxidative stress appears crucial in influencing the generation and aggregation of beta-amyloid plaques, offering a potential therapeutic route. Studies indicate that bolstering catalase activity may alleviate beta-amyloid-induced neurotoxicity, thus safeguarding against cognitive decline in experimental models, underscoring its neuroprotective capacity [52,53]. Data from a study implicate oxidative stress as a pivotal factor in AD etiopathology. Notably, mice expressing mitochondria-targeted antioxidant catalase (MCAT) exhibit reduced amyloid beta Aβ expression. MCAT intervention curbs abnormal amyloid precursor protein (APP) processing, reduces Aβ levels, and enhances Aβ-degrading enzyme activity in various disease stages in mice, suggesting that mitochondrial-targeted therapies could offer promising treatment avenues for AD [54]. The examination of plasma and erythrocytes from individuals with dementia of the Alzheimer type (DAT) showcases elevated Cu/Zn superoxide dismutase (Cu/Zn SOD) and catalase activity compared to non-DAT counterparts, underscoring potential compensatory neuroprotective responses in DAT individuals [55]. Utilizing the superoxide dismutase (SOD)/catalase mimetic EUK-207, the continuous treatment of 3xTg-AD transgenic mice elucidated the impact of oxidative stress on AD pathogenesis. This study revealed that long-term intervention mitigated beta-amyloid and tau pathologies, preserved cognitive function, and curbed brain oxidative stress, suggesting potential therapeutic benefits against AD-associated neurodegeneration and cognitive decline [56].

Parkinson’s disease (PD) is a progressive neurodegenerative disorder affecting the central nervous system and culminating in debilitating motor symptoms. The hallmark features of PD include impaired movement, tremors, muscular rigidity, postural instability, and compromised balance, attributable to the degeneration and demise of dopaminergic neurons within specific brain regions. The pathogenesis of PD has been strongly linked to several genetic factors, such as SNCA, LRRK2, DJ-1, PRKN (Parkin), PINK1, and GBA, underscoring the complex genetic underpinnings of the disease (https://www.ninds.nih.gov/health-information/disorders/parkinsons-disease accessed on 15 January 2024). Central to PD is the progressive loss of dopaminergic neurons within the substantia nigra (SN), leading to the characteristic motor impairments observed in the condition. Oxidative stress plays a significant role in Parkinson’s disease, marked by the accumulation of free radicals, lipid peroxidation, and impaired mitochondrial function. Within this context, catalase, localized in peroxisomes and mitochondria, is crucial in neutralizing hydrogen peroxide, a byproduct of cellular metabolism. The dysregulation of catalase activity may disrupt cellular redox homeostasis, exacerbating oxidative damage and hastening the degeneration of dopaminergic neurons in the substantia nigra [57,58,59].

Numerous research endeavors have explored the therapeutic potential of enhancing catalase activity as a neuroprotective strategy in managing Parkinson’s disease. Experimental studies employing catalase mimetics or gene therapy to boost cellular antioxidant defenses have shown promise in mitigating oxidative stress-related injury and preserving dopaminergic function, hinting at the potential utility of catalase modulation for slowing PD progression [60,61]. While the involvement of catalase in Parkinson’s disease pathogenesis is evident, further investigations are required to dissect the precise molecular mechanisms underlying catalase dysregulation in PD and to pinpoint targeted therapeutic avenues. A comprehensive comprehension of the interplay between oxidative stress and catalase activity in PD is pivotal for advancing our understanding of the disease pathophysiology and delineating potential therapeutic strategies [62,63].

Research findings demonstrating the neuroprotective effects of transduced PEP-1-catalase in preventing neuronal loss in PD animal models propose its potential as an effective treatment strategy for PD and other disorders linked to oxidative stress [64]. Additionally, investigations revealing catalase’s modulation of oxidative stress-induced channel activations underscore the enzyme’s crucial role in preserving mitochondrial function and neuronal viability, suggesting a significant impact on the pathogenesis of PD and associated sensory disturbances [65].

These insights into catalase’s therapeutic potential in neurodegenerative diseases like Alzheimer’s and Parkinson’s underpin the need for continued research and development of catalase-based therapies. These could offer new, effective treatment strategies for managing and potentially altering the course of these debilitating conditions.

### 5.2. Catalase and Cardiovascular Diseases

Cardiovascular disease encompasses a broad range of conditions affecting the heart and blood vessels, with coronary heart disease, exemplified by the accumulation of plaque leading to atherosclerosis, commonly equated with “heart disease.” Plaque buildup, comprising calcium, fat, cholesterol, and other blood components, narrows arteries, impedes oxygen-rich blood flow, and may cause angina or precipitate blood clots, leading to heart attacks. Catalase, pivotal in neutralizing reactive oxygen species (ROS) and averting oxidative damage, plays a protective role in combating endothelial dysfunction, atherosclerosis, and myocardial injuries in the context of cardiovascular diseases [59,66]. Observations of altered catalase activity in conditions like atherosclerosis and heart failure suggest a link between diminished antioxidant defenses, enhanced oxidative stress, and the progression of cardiovascular pathologies [67]. The imbalance between ROS production and antioxidant capacity, including catalase, contributes to lipid, protein, and DNA oxidative damage, exacerbating cardiovascular disease [68].

Explorations into enhancing catalase activity in cardiovascular disorders reveal promising avenues to combat vascular damage induced by oxidative stress and enhance cardiovascular function. Intervention strategies targeting catalase have shown protective effects against oxidative stress, highlighting the potential of catalase-based interventions in managing and treating cardiovascular diseases [69]. While catalase is a valuable factor in cardiovascular protection, translating these findings to clinical applications necessitates a thorough mechanistic understanding and targeted modulations of catalase activity in various cardiovascular conditions [70]. The intricate interplay between catalase, oxidative stress, and cardiovascular diseases underscores the significance of ongoing research to decode the nuances of antioxidant defense in preserving cardiovascular health [71]. Moreover, research on overexpressing catalase in myocytes has shown the ability to curb unfavorable cardiac remodeling and prevent the progression to heart failure in murine models [72].

In individuals with type 2 diabetes and diabetic cardiovascular disease, genetic polymorphisms in MnSOD, GPX1, and CAT genes have been linked to hypertriglyceridemia, suggesting the role of antioxidant defense in disease pathogenesis [73]. Notably, the overexpression of catalase in diabetic mouse hearts has demonstrated the downregulation of an ROS-dependent signaling pathway, thereby protecting against inflammatory reactions and protein nitration associated with diabetic cardiomyopathy, illustrating the potential cardioprotective properties of catalase [74]. Long-term type 1 diabetes appears to involve increased catalase and heme oxygenase-1 activity, potentially contributing to improved cardiac function [75].

### 5.3. Catalase and Diabetes Mellitus

Diabetes mellitus (DM) is a chronic metabolic disorder stemming from inadequate insulin secretion or impaired insulin utilization, leading to elevated blood glucose levels. The two primary types are type 1 diabetes mellitus (T1DM) and type 2 diabetes mellitus (T2DM), which are attributed to impaired insulin production and action [76]. Hyperglycemia, a hallmark of uncontrolled diabetes, instigates the generation of excess reactive oxygen species (ROS), culminating in oxidative stress. Catalase, a key enzyme safeguarding cellular redox equilibrium (Figure 2), counteracts hydrogen peroxide build-up, a critical facet in mitigating oxidative damage [77,78]. A deregulation of catalase levels in individuals with diabetes has been reported, with reduced activity observed in tissues like the pancreas and liver associated with hyperglycemic conditions [79,80].

The dysregulation of catalase activity may contribute to heightened oxidative stress, exacerbating insulin resistance and diabetes complications. Experimental studies focusing on enhancing catalase activity in diabetic models have demonstrated improved insulin sensitivity and curbed oxidative stress-related damage, suggesting the potential of catalase modulation as a strategy for managing diabetes complications [81,82]. Beyond mitigating oxidative stress, catalase’s role in preserving pancreatic beta cell function is vital. Beta cells, responsible for insulin secretion, are vulnerable to oxidative stress-induced dysfunction. Catalase’s action in neutralizing oxidative species can safeguard beta cells, maintaining insulin production and glycemic control [83].

Studies indicate that individuals with type 2 diabetes exhibit decreased blood catalase activity. This phenomenon, characterized by heightened hydrogen peroxide levels in muscle cells due to diminished blood catalase, is postulated to potentiate insulin signaling through oxidation-sensitive tyrosine phosphatase inactivation, impairing insulin receptor dephosphorylation [84]. Ratios of catalase/superoxide dismutase (SOD) and catalase/paraoxonase (PON) may correlate with poor glycemic control [85]. Alloxan-induced diabetes is linked to reduced blood catalase activity [86]. Investigations into overexpressing catalase (CAT) in Renal Proximal Tubular Cells (RPTCs) aimed at curbing hypertension and kidney damage in male offspring of diabetic mothers during infancy reveal promising protective effects via the Nrf2-HO-1 defense system activation [87]. Identifying a potential inverse correlation between catalase activity and type 2 diabetes risk underscores the importance of antioxidants like superoxide dismutase (SOD) and catalase in mitigating diabetes onset in specific populations [88]. Research evaluating salivary catalase and SOD levels in pregnant women with gestational diabetes reveals higher antioxidant enzyme levels, albeit not statistically significant, suggesting potential associations between antioxidant status and gestational diabetes [89].

### 5.4. Catalase and Rheumatoid Arthritis

Rheumatoid arthritis (RA) is a complex autoimmune disease characterized by joint inflammation and systemic manifestations. Oxidative stress and the role of antioxidants are crucial in the pathogenesis of RA. Studies have identified alterations in catalase activity in individuals with RA, pointing towards an imbalance between reactive oxygen species (ROS) production and antioxidant defenses, potentially exacerbating inflammation and joint damage in RA patients (Figure 2). Elevated levels of oxidative stress markers in the synovial fluid and tissues of RA patients suggest a dysregulation in oxidative balance contributing to disease progression [90,91]. Investigations into the therapeutic implications of catalase in RA have shown promising results in experimental models, indicating a potential avenue for therapeutic interventions by mitigating oxidative stress-related damage in synovial tissues and immune cells [92]. Furthermore, studies on antioxidant indicators such as thiol levels and catalase activity in early-stage RA patients have shown potential for assessing disease progression and understanding underlying mechanisms [93]. Research on patients with RA and osteoarthritis has revealed changes in various antioxidant parameters, highlighting the involvement of oxidative stress in these conditions. Genetic variations in antioxidant enzyme genes like catalase, superoxide dismutase, and tumor necrosis factor-alpha can also influence RA activity [94].

Moreover, investigations into antioxidant enzyme activities, including superoxide dismutase (SOD), glutathione peroxidase (GPX), and catalase in erythrocytes, along with plasma glutathione-S-transferase (GST) activity in RA patients, have provided insights into the role of oxidative stress in RA pathogenesis. The findings suggest an increased formation of oxygen free radicals, as evidenced by altered levels of malondialdehyde (MDA), glutathione (GSH), ascorbic acid, vitamin E, and catalase activity, emphasizing the significance of oxidative stress in RA development and progression [95]. In conclusion, understanding the complex interplay between oxidative stress, antioxidant enzymes such as catalase, and genetic variations in antioxidant-related genes is crucial in unraveling the mechanisms underlying rheumatoid arthritis and identifying potential therapeutic targets for disease management.

### 5.5. Catalase and Inflammatory Bowel Diseases (IBD)

Crohn’s disease and ulcerative colitis, collectively known as inflammatory bowel diseases (IBDs), present as relapsing and chronic conditions affecting the gastrointestinal tract, characterized by an unpredictable and progressive disease course. The global incidence of IBD has been on the rise over recent decades, resulting in a growing socioeconomic burden due to its chronicity, progressive nature, and high costs associated with its treatment [96]. Oxidative and nitrosative stresses have emerged as crucial pathophysiologic components in the development and progression of IBD. The release of cytokines and chemokines by inflammatory cells triggers oxidative stress during inflammation, leading to the overproduction of reactive oxygen species (ROS) [97].

Studies focusing on peripheral blood T cells from IBD patients and healthy individuals have revealed significant insights. Reduced catalase activity in T cells from patients with Crohn’s disease was associated with inhibited apoptosis, suggesting impaired cellular function. This highlights the potential importance of catalase in regulating proper T-cell behavior and its implications for IBD pathogenesis [98]. The development of an artificial enzyme, Sul-MPBs, with catalase, superoxide dismutase (SOD), and glutathione peroxidase (GPx)-like activities demonstrates promising ROS-scavenging capabilities and protection against hydrogen peroxide-induced mitochondrial dysfunction in vitro [99].

As a vital antioxidant enzyme, Catalase plays a crucial role in neutralizing oxidative stress by converting hydrogen peroxide into harmless byproducts. Alterations in catalase activity observed in individuals with IBD suggest a potential imbalance in antioxidant defenses, warranting further investigation into the therapeutic strategies targeting catalase to mitigate oxidative damage and inflammation in the gastrointestinal mucosa [100,101].

Researchers have explored the impact of propolis on catalase activity in colonic tissue, an essential antioxidant enzyme responsible for hydrogen peroxide breakdown. While some studies did not observe significant enhancements in catalase activity with propolis treatment, one study reported a significant increase in catalase levels following propolis administration, suggesting a potential beneficial effect of propolis in bolstering antioxidant defenses in the context of IBD [102]. Further elucidation of the mechanisms underlying catalase dysregulation in IBD and its implications for disease progression is crucial for the development of targeted therapeutic approaches to manage IBD effectively.

### 5.6. Catalase and Cancer

In cancer, disrupting the delicate balance between reactive oxygen species (ROS) generation and antioxidant defenses, including catalase, contributes to DNA damage and genomic instability. Investigations into the dual role of catalase in cancer have revealed that while its protective function in normal cells is essential, it may inadvertently support cancer cell survival. The intricate mechanisms underlying catalase in cancer shed light on potential therapeutic strategies targeting the interplay between oxidative stress and cancer progression, highlighting the importance of understanding catalase’s nuanced involvement in cancer biology for the development of targeted interventions [103,104]. Research on catalase core promoters has identified the presence of CCAAT boxes and GGGCGG motifs, rich in GC residues and located close to the catalase promoters’ upstream region. Transcription factors Sp1 and NF-Y are proposed to positively regulate catalase production by interacting with these elements in the core promoter; these factors control catalase expression in cancer cells [105].

Histological evaluations revealed enhanced resistance to oxidative stress induced by hydrogen peroxide in a hybrid model involving MMTV-PyMT transgenic mice with metastatic breast cancer expressing the human catalase gene (mCAT). They decreased p38MAPK activity in the lung tissues, suggesting a link between ROS signaling and p38MAPK pathways [106]. A catalase-producing L. lactis strain inhibited tumor development in an experimental DMH-induced colon cancer model, indicating a potential therapeutic approach for cancer treatment [107]. Patients with bladder cancer exhibited significantly higher serum levels of carbonic anhydrase, catalase, and adenosine deaminase compared to controls, indicating potential biomarkers for bladder cancer diagnosis and progression [108].

Catalase has also been linked to the regulation of apoptosis, a programmatic cell death process essential for maintaining tissue equilibrium. While primarily recognized for its role in detoxifying hydrogen peroxide and ameliorating oxidative stress, catalase has shown involvement in cellular signaling pathways associated with apoptosis. Research indicates that catalase can influence apoptotic pathways by modulating redox balance, impacting the cellular sensitivity to apoptotic signals. Altered catalase activity under specific conditions has been correlated with the dysregulation of apoptotic processes, affecting cell survival and contributing to disease progression. The complex interplay between catalase and apoptosis underscores the enzyme’s broader functional significance beyond its antioxidant capabilities. It provides avenues for the further investigation into cellular destiny and formulating therapeutic strategies for conditions where apoptosis is pivotal [109].

Studies have demonstrated that catalase overexpression diminishes smooth muscle growth and accelerates apoptosis, potentially via a mechanism involving COX-2 [110]. Further evidence of catalase’s proapoptotic activity has been suggested, indicating that H_2_O_2_ is a critical regulator of both apoptosis and stress responses while supporting the antiapoptotic impact of heat shock proteins in myeloid cells [111]. UV-induced apoptosis in keratinocytes involves intricate molecular mechanisms, with the intrinsic pathway encompassing DNA damage and generating reactive oxygen species (ROS). Catalase overexpression protects cells against UVB exposure by preventing DNA damage from late ROS elevation [112]. Additionally, Sirt1, an NAD+-dependent protein deacetylase that governs apoptosis, has been implicated in interacting with catalase. Elevated Sirt1 levels, concomitant with increased catalase expression, were observed when H_2_O_2_ was used to induce apoptosis. The upregulation of catalase through Sirt1 overexpression was found to avert H_2_O_2_-induced apoptosis, highlighting the interconnected roles of these proteins in regulating cell death [113].

### 5.7. Catalase and Chronic Obstructive Pulmonary Disease (COPD)

Chronic obstructive pulmonary disease (COPD) remains one of the most prevalent chronic illnesses, significantly impacting morbidity and mortality rates. The pathophysiology of COPD is characterized by an imbalance between antioxidants and oxidants, known as oxidative stress, which is a critical factor in this inflammatory lung disease. Oxidative stress, alongside systemic and persistent localized inflammation, is a hallmark feature of COPD [114]. The increased production of reactive oxygen species (ROS) from inflammatory cells and exposure to oxidants from inhaled sources contribute to the elevated oxidative stress observed in COPD, potentially leading to an exacerbated inflammatory response and the generation of lipid peroxidation products [115]. Oxidative stress is now recognized as a primary pathogenic component in COPD, accelerating disease progression and severity by playing a crucial role in initiating and perpetuating the lung’s inflammatory response [116]. Studies examining COPD patients have revealed significant alterations in antioxidant status, particularly catalase (CAT) activity, suggesting its potential role in disease pathophysiology [117].

Reductions in catalase levels at both the mRNA and protein levels in the bronchiolar epithelium of smokers with COPD highlight a correlation between decreased bronchiolar catalase and COPD development, with the regulation of catalase being crucial in preventing cell damage induced by cigarette smoke exposure [118]. Evidence of altered catalase activity in COPD patients further supports the connection between impaired antioxidant defenses and disease progression [119]. The dysregulation of catalase observed in COPD patients may contribute to the oxidative burden present in this condition, influencing the advancement of airway inflammation and decline in lung function. Exploring approaches to enhance catalase activity may offer valuable insights into potential therapeutic strategies to mitigate oxidative stress and alleviate COPD-associated lung damage [120].

In a study involving COPD patients experiencing severe exacerbations, measurements of superoxide dismutase (SOD) and catalase (CAT) activities in serum, exhaled breath condensate, and sputum samples were conducted. Patients with acute exacerbations of COPD exhibited significantly higher SOD and CAT activities compared to stable COPD patients, emphasizing the potential role of these antioxidant enzymes in COPD exacerbations [121]. The examination of catalase and superoxide dismutase gene polymorphisms and functional activity in smokers with COPD revealed no significant variations in allele frequencies or genotypes between patient and control groups, suggesting a potential role of erythrocyte catalase activity in distinguishing COPD patients from healthy individuals [122].

### 5.8. Catalase and Hepatic Disease

The liver plays a crucial role in various physiological functions, including metabolism, the storage and release of glucose, the synthesis of proteins, and bile secretion for aiding digestion [123]. Iron homeostasis, xenobiotic detoxification, gluconeogenesis, lipid metabolism, and storage are among the essential processes orchestrated by the liver. It also contributes to the production of significant plasma proteins and plays a vital role in processing hemoglobin and converting toxic ammonia into urea. Chronic liver disease denotes the progressive deterioration of liver function [124].

The liver’s functions encompass bile excretion, the detoxification of metabolic byproducts, and the synthesis of clotting factors and other essential proteins. Fibrosis and cirrhosis, outcomes of a continual process involving liver inflammation, destruction, and regeneration, mark advanced stages of chronic liver disease. Disruptions to liver architecture, the formation of extensive nodules, vascular restructuring, neo-angiogenesis, and extracellular matrix deposition typify cirrhosis. Hepatic stem cells are pivotal for liver tissue regeneration, while stellate cells and fibroblasts play a crucial role in the onset of fibrosis and cirrhosis at the cellular level [125].

Alterations in catalase activity in hepatic disorders indicate a potential association between impaired antioxidant defenses and liver pathologies. Investigations focusing on non-alcoholic fatty liver disease (NAFLD) have revealed a dysregulation of catalase linked to oxidative stress and liver damage, suggesting that modulating catalase levels could hold therapeutic potential in combating oxidative stress-induced liver injury and providing avenues for hepatic disease management. Delving into the mechanisms behind catalase dysregulation in various hepatic conditions is essential for formulating targeted approaches to fortify antioxidant defenses and uphold liver function [126].

In specific conditions such as acute alcoholic hepatitis, decompensated cardiac and circulatory failure, and fatty liver, elevated serum catalase activity has been reported [127]. Research utilizing in vivo models has demonstrated the protective role of endogenous catalase in the early stages of non-alcoholic fatty liver disease by preserving liver redox balance and shielding against hepatic injury induced by high-fat diets [128]. Furthermore, a study involving children with chronic cholestatic liver disease highlighted significantly higher levels of superoxide dismutase (SOD), glutathione peroxidase (GPx), and catalase in the hepatic tissues of affected children compared to controls. Patients with extrahepatic biliary atresia exhibited elevated enzyme levels across the board, while those with neonatal hepatitis and intrahepatic bile duct paucity showed significant increases in GPx and CAT enzymes [129].

### 5.9. Catalase and Chronic Kidney Disease (CKD)

Chronic Kidney Disease (CKD) is characterized by a progressive decline in kidney function over time, often leading to complications such as hypertension, fluid retention, and electrolyte imbalances [130]. Oxidative stress is a recognized contributor to CKD pathogenesis, with antioxidant enzymes like catalase crucial for maintaining redox balance within the kidneys (Figure 2). Studies have demonstrated alterations in catalase activity in individuals with CKD, hinting at a potential association between impaired antioxidant defenses and the advancement of renal damage [131]. In a chronic renal failure (CRF) study involving rats, a significant downregulation of immunodetectable catalase and glutathione peroxidase proteins was observed in the remaining kidney of the CRF group. While glutathione peroxidase activity was relatively unaffected, catalase activity in the remaining kidney was significantly reduced [132].

Another study evaluating the impact of exercise on oxidative stress markers in male Wistar rats revealed notable increases in creatinine and urea levels, superoxide generation, antioxidant enzymes (catalase and superoxide dismutase), and oxidative damage in CKD groups compared to sham-treated animals [133]. Comparative analysis among end-stage renal failure (CRF) patients, hemodialysis (HD) patients, and peritoneal dialysis (PD) patients showed variations in antioxidant enzyme activity levels. Plasma superoxide dismutase (SOD) activity was lower in HD compared to CRF and PD patients. In contrast, glutathione peroxidase activity (GSH-Px) was reduced in both HD and PD groups compared to CRF. Only PD patients exhibited decreased catalase activity compared to CRF [134]. A study on patients with Chronic Kidney Disease measured various parameters, including serum urea, creatinine, uric acid, superoxide dismutase (SOD), catalase, malondialdehyde (MDA), total antioxidant capacity (TAC), and creatinine. The findings revealed significantly increased MDA levels and reduced TAC, SOD, and catalase levels in CKD patients with and without hypertension, indicating abnormal renal functions [135].

### 5.10. Catalase and Inflammation

Catalase is vital in maintaining redox balance within cells and tissues, preventing the excessive accumulation of reactive oxygen species (ROS) that could lead to inflammatory damage. Research suggests that changes in catalase activity can impact the inflammatory response, with decreased catalase levels associated with elevated inflammation in certain conditions. Moreover, catalase’s anti-inflammatory properties extend beyond its antioxidant function, as it has the potential to influence signaling pathways involved in inflammatory cascades directly. The therapeutic potential of catalase in regulating inflammation is significant, with implications for various diseases such as autoimmune disorders, cardiovascular conditions, and neurodegenerative diseases. Investigating the intricate interplay between catalase and inflammatory pathways offers promise for developing targeted therapeutic strategies to modulate inflammatory responses and mitigate the effects of chronic inflammation on overall health [136].

Mitochondrial dysfunctions are known to be programmable based on mechanisms related to intracellular inflammation processes. Mitochondria-targeted catalase has shown promise in improving cardiovascular, neurological, metabolic, and immune-related conditions by addressing inflammation-related pathologies [137]. The local administration of liposome-encapsulated catalase and superoxide dismutase has been reported to reduce periodontal inflammation in beagles [138].

Additionally, a superoxide dismutase/catalase mimic called MnTMPyP has shown efficacy in reducing inflammatory markers in cases of acute kidney injury induced by ischemia, leading to decreased levels of creatinine, interleukins (IL-2, IL-4, IL-13), tumor necrosis factor-alpha (TNF-α), and inhibiting macrophage (ED1+) and T lymphocyte (CD8+) infiltration [139]. Studies by Mossman and colleagues have shown that polyethylene glycol-conjugated catalase can inhibit lung injury, inflammation, and interstitial pulmonary fibrosis in a model of asbestosis-induced lung injury [140]. Conversely, bacterial catalase and superoxide dismutase have promoted inflammation due to their ability to neutralize ROS and generate pro-inflammatory cytokines [141].

### 5.11. COVID-19 and Catalase

The higher prevalence of COVID-19 in individuals with non-communicable diseases, particularly those over 60 and those with chronic conditions like diabetes, asthma, and heart or respiratory conditions, has been noted in various studies [142]. Diabetes, in particular, has been suggested to play a causal role in the severity of COVID-19 and subsequent SARS-CoV-2 infection [143]. Research involving 150 healthy volunteers and 600 COVID-19 patients, including severe and non-severe cases, revealed disturbances in the oxidant-antioxidant balance in COVID-19 patients, favoring oxidant indicators [144].

Studies on critically ill COVID-19 patients with varying ICU stays highlighted increased lipid peroxidation and deficiencies in antioxidants, trace elements, and other oxidative stress markers, indicating a significantly altered state of systemic oxidative stress in these patients [145]. The assessment of malondialdehyde (MDA), total oxidant status (TOS), catalase (CAT) activity, and superoxide dismutase (SOD) in 48 individuals revealed significantly higher CAT activity in COVID-19 cases compared to controls, with no significant differences between ICU and non-ICU groups [146]. The further analysis of blood samples from recovered COVID-19 patients and individuals vaccinated with Sputnik V showed that COVID-19 survivors had the highest catalase activity in their blood IgGs compared to vaccinated individuals and healthy donors. This suggests that COVID-19 infection may lead to the generation of antibodies with increased catalase activity, potentially posing risks with elevated hydrogen peroxide breakdown [147].

In another study, COVID-19 patients exhibited a reduced total antioxidant capacity but higher levels of antioxidant enzymes (superoxide dismutase and catalase) and markers of oxidative cell damage (carbonyl and lipid peroxidation) compared to controls, indicating an imbalanced redox status in these individuals [148].

### 5.12. Catalase and Reproductive Health

Reproductive health is influenced by various factors, among which oxidative stress plays a crucial role. Oxidative stress has been linked to male and female infertility, as well as complications during pregnancy. Ensuring proper antioxidant defenses, including enzymes like catalase, is essential for maintaining reproductive health [149]. In males, oxidative stress can lead to sperm DNA damage, reduced motility, and impaired function, contributing to infertility. Antioxidants such as catalase are known to mitigate oxidative damage to sperm, potentially enhancing fertility outcomes in men [150].

In females, conditions like endometriosis, polycystic ovary syndrome (PCOS), and preeclampsia have been associated with oxidative stress, impacting reproductive health and pregnancy outcomes [151]. ROS are critical signaling molecules in physiological processes and pathological events affecting the female reproductive system. Oxidative stress influences various physiological processes, including egg development, fertilization, embryo development, and pregnancy. Studies indicate that oxidative stress may play a role in preventing age-related decline in fertility, while conditions like preterm labor and ovarian cancers are also linked to ROS-mediated processes [151,152].

Antioxidants, notably catalase, can alleviate oxidative stress and inflammation in these conditions, potentially improving fertility and pregnancy outcomes [153]. While research on catalase in reproductive health is still evolving, its role in combating oxidative stress suggests potential benefits in maintaining fertility and supporting healthy pregnancies. However, further studies are warranted to fully comprehend the specific impact of catalase on reproductive health and its therapeutic applications [154,155].

Analytical studies comparing menopausal and reproductive women have shown significant differences in catalase enzyme levels, indicating variations in catalase activity between these groups [156]. Moreover, obese women with and without polycystic ovary syndrome (PCOS) have exhibited notably increased catalase activity in follicular fluid compared to non-obese women [157]. In an in vivo study focusing on folliculogenesis, it was observed that catalase activity decreased with follicle size. At the same time, total antioxidant capacity (TAC) exhibited an inverse trend, implying a significant role of catalase as a primary antioxidant defense in early follicle development [158].

### 5.13. Anti-Microbial Properties of Catalase

For intracellular bacteria to avoid neutrophil phagocytosis, catalase plays a crucial virulent role [159]. However, catalase is a crucial defense mechanism against bacterial infections by neutralizing reactive oxygen species (ROS) generated during the infectious process. Endogenous catalase produced by the host protects tissues from oxidative damage induced by bacterial-triggered ROS, regulates immune responses to prevent excessive inflammation, and aids phagocytes in killing engulfed bacteria [62]. Conversely, certain bacteria can produce catalase to evade host immune defenses and survive in hostile environments. Strategies involving catalase inhibitors have been explored as potential antibacterial agents to enhance bacterial susceptibility to oxidative stress and traditional antibiotics [160]. Consequently, catalase assumes a multifaceted role in the host–pathogen interaction, influencing both host defense mechanisms and bacterial survival tactics during infection [141,161].

In a study utilizing the catalase inhibitor 3-amino-1,2,4-triazole (3-AT), the inhibition of catalase was investigated to ascertain the impact on Brucella abortus intracellular proliferation within RAW 264.7 macrophage cells and an ICR mice model during Brucella infection. The findings revealed that 3-AT effectively inhibited the growth of B. abortus within macrophages. The reductions in TNF-α, IL-6, and IL-10 levels and alterations in the CD4+/CD8+ T-cell ratio indicated a dampening immune response to the infection. These results underscore the immunomodulatory and protective attributes of 3-AT (catalase inhibitor) in combatting Brucella infection [162,163].

**Figure 5:**
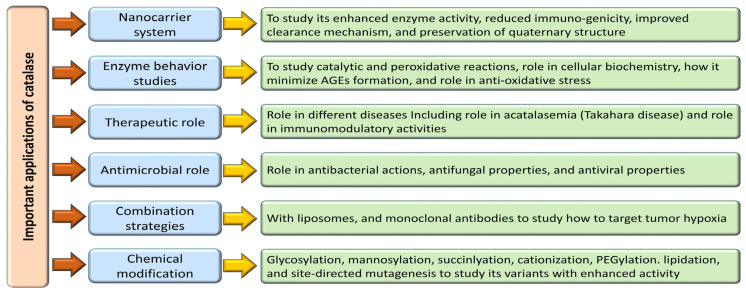
Multifaceted role of catalase in clinical, biochemical, and other biological studies.

Catalase exhibits antifungal effects primarily by producing reactive oxygen species (ROS) and breaking down hydrogen peroxide (Figure 5). These ROS damage fungal cell structures and impair cell functions, which is particularly effective against major fungal pathogens such as Candida albicans, Aspergillus fumigatus, and Cryptococcus neoformans. These fungi, known for their severe infections and resistance to standard therapies, suggest that catalase could be crucial in developing new antifungal treatments [164]. Clinically, catalase has been successfully combined with traditional antifungal agents to combat drug-resistant infections, showing promise for enhancing treatment effectiveness [165]. Incorporating catalase with azole antifungals also opens new avenues for advancing antifungal regimens. Experiments with Saccharomyces cerevisiae have revealed that changes in catalase activity can affect cell survival under antifungal stress. Adjusting catalase levels via genetic or biochemical methods modifies yeast cell responses to azoles. This points to a sophisticated relationship between fungal responses to oxidative stress and antifungal drug resistance [166].

Studies also show that modifying catalase expression in *C. albicans* significantly impacts its ability to withstand oxidative and combined stresses, affecting survival in adverse conditions. Although increased catalase expression boosts resistance to oxidative stress, it also challenges iron regulation, adversely affecting fungal health in environments with limited iron [167]. The diverse role of catalase in fungal biology and its interactions with antifungal agents provide valuable perspectives for enhancing antifungal treatments. Catalase, a ubiquitous antioxidant enzyme in many living organisms, has been identified as pivotal in modulating viral infections (Figure 5). Empirical studies suggest that catalase exhibits specific antiviral effects against a variety of viruses, including herpes simplex virus (HSV), human immunodeficiency virus (HIV), and hepatitis C virus (HCV) [168]. A primary mode of action by which catalase exerts its antiviral activity is the decomposition of hydrogen peroxide (H_2_O_2_), a reactive oxygen species instrumental in the replication process of many viruses. By catalyzing the conversion of H_2_O_2_ into water (H_2_O) and oxygen (O_2_), catalase interferes with the oxidative environment needed for effective viral replication within host cells [169].

Additionally, catalase has been observed to modulate the inflammatory response during viral infections. It achieves this by attenuating the production of pro-inflammatory cytokines, critical mediators in the pathogenesis and progression of viral diseases such as HIV. This enzymatic activity not only limits inflammation but also regulates lipid peroxidation and boosts the activity of other antioxidant enzymes, including superoxide dismutase (SOD) and catalase itself, further amplifying its antiviral effects [170]. In the context of respiratory viruses, catalase shows potential in attenuating pulmonary fibrosis—a significant complication in diseases such as COVID-19 caused by the SARS-CoV-2 virus. This suggests an important therapeutic role for catalase in managing and potentially mitigating the effects of COVID-19 by inhibiting specific pathological processes involved in severe cases [171].

### 5.14. Immunomodulation Properties of Catalase

Catalase plays a crucial role in immunomodulation by controlling reactive oxygen species (ROS) levels by neutralizing hydrogen peroxide (H_2_O_2_). This function is essential for maintaining cellular redox equilibrium and influencing immunological responses [172]. In inflammatory conditions, catalase activity reduces the excess formation of ROS, thereby mitigating tissue damage and inflammation associated with chronic inflammatory diseases and autoimmune disorders (Figure 5). Moreover, catalase has been shown to modulate immune cell function, such as macrophage polarization and T cell activation, by regulating intracellular ROS levels [173]. The regulation of ROS by catalase can impact cytokine and chemokine production, thereby shaping the overall immune response to pathogens and antigens. As a critical immunomodulatory enzyme, catalase contributes significantly to fine-tuning immune responses and maintaining immune homeostasis.

In chronic autoimmune diseases like rheumatoid arthritis (RA), an overabundance of pro-inflammatory (M1) macrophages and hyperactive immune cells (HICs) in the joint synovium, along with increased ROS concentrations, is commonly observed. An immunomodulatory nanosystem (IMN) proposed for RA therapy aims to modulate and restore HIC activity in inflamed tissues [174]. In a 2013 study, the immunomodulatory effects of recombinant human catalase were evaluated in an H1N1-infected mouse model at doses of 50 and 100 kU/mice/day. This study showed that recombinant human catalase could dose-dependently enhance compromised phagocytosis, alleviate organ weight reductions in the spleen and thymus, and significantly reduce lung tissue viral load [175]. Furthermore, catalase nano-complexes like CAT-Ce6@OMV-aPDL1 are utilized in oxygenated photodynamic treatment (PDT) and immunotherapy for tumors, demonstrating significant antitumor effects and offering new possibilities for combinatorial therapy against various types of cancer [176].

### 5.15. Catalase and Advanced Glycation End Products

Advanced glycation end products (AGEs) are detrimental compounds that develop through the non-enzymatic interaction between reducing sugars and biomolecules such as proteins, lipids, or nucleic acids. Implicated in diverse pathologies, including diabetes, cardiovascular diseases, and neurodegenerative disorders, AGEs pose a significant health concern [177] (Figure 5). In this complex landscape, catalase emerges as a potential player in mitigating the formation and impact of AGEs. Catalase’s functionality in preventing oxidative stress is paramount. It is achieved by breaking hydrogen peroxide, a precursor to reactive oxygen species (ROS) formation. By reducing oxidative stress, catalase indirectly holds promise in minimizing AGE formation. Moreover, the antioxidant properties inherent to catalase offer direct protection against AGE-induced damage by scavenging ROS molecules [178,179,180]. The multifaceted interplay between catalase and AGEs beckons further exploration to unravel the specific mechanisms through which catalase influences AGE formation and its downstream effects. This deeper understanding is essential for delineating the full therapeutic potential of catalase in combating Age-related diseases and paving the way for targeted interventions in this critical area of research.

## 6. Current Limitations of Catalase Enzyme for Therapeutic Applications

The therapeutic potential of catalase as an antioxidant therapy has garnered significant interest in the scientific community. However, despite its promise of mitigating oxidative stress, the practical application of catalase in therapeutics is encumbered by several noteworthy limitations. One primary challenge resides in catalase’s short half-life, poor cellular absorption, and the inherent difficulty in safely and effectively transporting the enzyme into tissues and systems like the central nervous system [181]. Key hurdles associated with using catalase in therapeutic settings include determining the optimal dosage, developing efficient delivery methods, and ensuring tissue specificity. Additionally, considerations such as production costs and ethical concerns regarding the enzyme’s sourcing present obstacles to its widespread application. Moreover, the complex interplay of catalase with endogenous systems necessitates a comprehensive understanding of potential unintended consequences in therapeutic contexts [182].

Further complicating its application are challenges related to catalase’s stability under harsh conditions, limited cellular uptake, and the potential for immunogenic responses, all of which can impact its efficacy and safety profile. Notably, catalase’s specificity for hydrogen peroxide poses constraints in conditions where other reactive oxygen species contribute to oxidative stress, necessitating strategies for more comprehensive targeting approaches [183]. Mitigating these limitations requires a concerted interdisciplinary effort coupled with technological advancements to fully unleash catalase’s therapeutic potential in diseases associated with oxidative stress. Addressing these challenges through innovative research and technological breakthroughs is crucial for maximizing catalase’s effectiveness and safety in combatting oxidative stress-related conditions.

## 7. Modulation of Catalase Activity through Various Strategies

Enzymes hold tremendous potential as therapeutic agents, yet their practical application is hindered by challenges in effective delivery, necessitating the development of suitable nanocarrier systems [184]. Extensive studies on the interplay between enzymes and nanocarriers have revealed enhanced enzymatic activity, reduced immunogenicity, and improved clearance mechanisms. Preserving the quaternary structure of enzymes within these carriers maintains their catalytic function and optimizes their encapsulation efficiency, thereby facilitating their therapeutic availability [184,185]. Nanocarriers come in diverse compositions, topographies, and shapes, with nanostructures like erythrocytes, liposomes, nanoparticles, virosomes, and extracellular vesicles emerging as critical platforms for coupling with macromolecules for diagnostic and therapeutic purposes [69,184,185,186,187]. Catalase, a pivotal enzyme with diverse therapeutic applications, has demonstrated efficacy in animal models ranging from tumor scenarios to hepatic injuries thanks to innovative delivery strategies such as galactosylation, mannosylation, succinylation, cationization, and PEGylation [188]. Notably, combining catalase with liposomes and monoclonal antibodies has shown promise in targeting tumor hypoxia effectively, thereby enhancing immunotherapeutic outcomes against melanoma [189]. In a different approach, catalase-loaded poly (lactic-co-glycolic acid) nanoparticles have emerged as a potent tool in shielding human neurons from oxidative damage, exhibiting high encapsulation efficiency and sustained antioxidant effects, highlighting their potential for neural protection [181].

Nanoparticles within the 10–1000 nm size range, known as solid dispersion particulates, offer advantages such as enhanced particle mobility, diffusion, thermal stability, storage capacity, increased surface area, and catalytic activity modulation, which are notably beneficial for enzymes like catalase used in antioxidant therapy [190]. Overcoming the challenges posed by the unstable nature and poor delivery of catalase, nanotechnology-based strategies have emerged as practical solutions for safeguarding human neurons from oxidative damage and shielding against H_2_O_2_-induced oxidative injury [181]. Efforts to further enhance enzyme stability, catalytic efficiency, and bioavailability through nanotechnology hold promise for advancing therapeutic outcomes in enzyme-based treatments.

The interaction between SiO_2_ nanoparticles, catalase (CAT), and human mesenchymal stem cells (hMSCs) was meticulously investigated by Mousavi and colleagues in 2019 through a comprehensive approach involving cellular assays, molecular docking, dynamics studies, and various spectroscopic techniques. Their findings indicated that SiO_2_ nanoparticles at low concentrations exhibit minimal impact on catalase or hMSCs, suggesting their potential utility as carriers for delivering catalase to hMSCs [191]. Notably, the catalytic activity of silica nanoparticles and catalase hybrids in decomposing hydrogen peroxide mirrored that of freely diffusing enzymes in solution, underscoring their efficacy in catalytic processes [192].

Innovative strategies leveraging cell-based delivery methods, such as stem cell treatment and customized cell carriers, have been devised to facilitate the targeted delivery of catalase to specific tissues [193]. Engineered cells capable of expressing catalase offer extended tissue residency and sustained therapeutic effects, serving as living bioreactors for continuous enzyme production. Recent advancements in protein engineering, chemical modification, and nanotechnology have enhanced catalase stability, activity, and bioavailability. Techniques like site-directed mutagenesis and directed evolution enable the creation of catalase variants with improved catalytic efficiency [183].

Various chemical modifications such as PEGylation, glycosylation, and lipidation have enhanced its stability and bioavailability to safeguard catalase from enzymatic degradation and immune recognition. Surface modifications using stealth polymers like PEG mitigate immunogenic responses, prolong circulation duration, and heighten treatment efficacy [194,195,196,197,198]. Additionally, nanotechnology-based approaches, including encapsulating catalase in protective matrices and immobilizing it, offer a conducive environment for maintaining enzyme activity and enabling controlled release at targeted sites. Nanostructured materials like carbon nanotubes, graphene, and metal–organic frameworks serve as ideal platforms for catalase immobilization, enhancing therapeutic outcomes with regulated enzyme delivery [193,198,199,200]. Table 1 shows various studies providing evidence for the modulation of catalase.

## 8. Utilizing Catalase in Combination Therapies for Enhanced Antioxidant Capacity

A synergistic approach involving catalase in combination with other antioxidants has shown promise in enhancing antioxidant capacity and mitigating oxidative stress-induced damage. Rather than solely relying on supplemental antioxidants like vitamins C and E, which possess limited scavenging capacity, a more efficacious strategy, as proposed by Nelson and collaborators, involves the modest induction of catalytic antioxidants such as superoxide dismutase (SOD) and catalase [203]. This method presents a more sustainable antioxidant defense mechanism that can effectively combat the oxidant burden.

Maksimenko (2005) investigated the potential of covalently conjugated formulations, superoxide dismutase-chondroitin sulfate, and catalase-chondroitin sulfate for intravenous administration. Notably, the enzymic covalent conjugate of superoxide dismutase-chondroitin sulfate-catalase exhibited significantly reduced antithrombotic effects compared to individual formulations, showcasing the synergistic benefits of combining SOD and catalase [204]. In a study involving A549 cells in exponential growth, the administration of catalase either alone or in conjunction with chemotherapeutic agents (hydroxyurea, paclitaxel, daunorubicin, 5-fluorouracil, and cisplatin) was evaluated. Notably, combining catalase (or catalase analogs) with conventional chemotherapy drugs, particularly cisplatin, emerged as a promising therapeutic strategy for lung cancer treatment [205]. This combinatorial approach holds the potential for enhancing treatment efficacy by leveraging the antioxidant properties of catalase in synergy with conventional chemotherapeutic interventions, offering a multifaceted therapeutic approach against lung cancer. Table 2 shows various combination studies for enhancing the antioxidant activities of catalase.

## 9. Future Perspectives of Catalase Enzyme

The prospects of the catalase enzyme are poised for significant advancements driven by innovations in nanotechnology, gene therapy, and protein engineering. By integrating catalase into nanoparticle-based delivery systems, exploiting gene-editing tools, and harnessing protein engineering techniques, catalase’s stability, bioavailability, and targeted delivery for therapeutic purposes can be substantially augmented. Customizing catalase variants to cater to specific therapeutic requirements, investigating potential synergies with other antioxidants, and utilizing computational models for predictive enzyme design represent promising avenues for optimizing the therapeutic efficacy of catalase [43,183].

Exploring the potential utility of catalase as a diagnostic or prognostic biomarker in conditions characterized by oxidative stress is an emerging frontier in research. Embracing sustainable sourcing practices, conducting rigorous clinical trials, and fostering educational initiatives are pivotal for translating catalase innovations into tangible clinical applications. Moreover, in neurodegenerative disease research, the neuroprotective attributes of catalase present a compelling area for future investigations, potentially offering novel therapeutic avenues [43,183].

The multifaceted and dynamic future perspectives of catalase underscore its critical role in driving personalized medicine initiatives, enhancing disease prevention strategies, and ultimately contributing to improved healthcare outcomes. By embracing cutting-edge technologies and exploring novel applications, catalase stands at the forefront of innovative approaches to revolutionize therapeutic interventions and promote holistic healthcare solutions for diverse patient populations [43,183].

## 10. Peroxiredoxins

The universal family of cysteine-dependent peroxidase enzymes known as peroxiredoxins (Prxs) is primarily responsible for controlling the amounts of peroxide in cells. These enzymes exhibit a remarkable diversity of changes in their oligomeric forms and vulnerability to control by hyperoxidative inactivation and other post-translational modifications. They are frequently present at high levels and have the ability to swiftly remove peroxides [207].

Prxs effectively scavenge organic and H_2_O_2_, shielding cells from the buildup of ROS and contributing to redox-dependent signaling pathways. This family of enzymes has six isoforms in mammalian cells. Although they are expressed in a variety of organs and cell types, peroxiredoxins are broadly distributed. Although it is present in other tissues, the kidneys, liver, and lung exhibited the highest levels of Prx1 expression. Prx4 is more heavily expressed in the pancreas, spleen, liver, testis, and lungs than in other organs. In contrast, Prx2, Prx3, and Prx5 contain enzymes that are widely distributed. The lung contained the greatest amounts of peroxiredoxin 6, which was also present in the brain, heart, liver, spleen, kidney, and testis [208]. Prxs had been determined to be ~1000 times slower than catalase and glutathione peroxidases (Gpxs). It was devoid of common redox centers like heme, flavin, selenocysteine, or metals [209].

The resistance of malignancies and cancer-derived cell lines to specific chemo- and radiotherapies has been linked to high Prx levels. Prxs have also been linked to aberrant nitration in early Alzheimer’s patients, as well as to a role in inducing inflammation linked to ischemic brain injury. Furthermore, infections are attractive candidates for the creation of innovative antibiotics since they depend on their Prxs to elude host immune systems. The action of Prxs, which are cysteine-based peroxidases, is independent of specific cofactors [209]. Peroxide levels and peroxidases interact intricately since most eukaryotes have numerous Prx isoforms in addition to other peroxidases like catalase and Gpxs. Understanding the functions Prxs play in cellular homeostasis, drug resistance in cancer development, Alzheimer’s disease, and ischemic brain injury will require examining these issues and deciphering these intricate relationships [207].

## 11. Supplementation of Small-Molecule Antioxidants and Their Negative Effects

Oxidative stress plays a major role in various diseases. The management of oxidative stress-induced diseases has increased interest in the use of antioxidant supplements. Increased levels of antioxidants by supplementation have been proposed as a way to prevent, delay, or improve the effects of these diseases associated with oxidative stress. Supplements containing dietary vitamins, such C and E, are also an option [210]. Antioxidant supplementation is still debatable, though, as some research suggests negative outcomes. More recent research refutes the notion that antioxidant supplements are good for human health [211].

This idea is supported by a large number of studies, while other studies find little evidence that treating cell lines with antioxidant supplements lowers the chance of cancer development. Comparable results were noted in observational research evaluating a possible correlation between antioxidant supplementation and cancer risk. Interestingly, a number of epidemiological studies even revealed a possible link between antioxidant supplement users and an increased risk of cancer. However, the inability of these studies to assess the temporal relationship between oxidative stress and tumor initiation or take confounding factors into account limited their scope [212].

The Alpha-Tocopherol, Beta-Carotene (ATBC) trial showed that using BC supplements was linked to an 18% higher chance of developing lung cancer. Furthermore, individuals receiving BC supplementation had an 8% higher all-cause mortality rate, with ischemic heart disease and lung cancer accounting for the majority of deaths [213]. The ATBC subjects had a 20% increased risk of dying from prostate cancer [214]. The first study to examine the impact of vitamin A supplementation in preventing lung cancer was the Carotene and Retinol Efficacy Trial (CARET) prior to ATBC [215].

## 12. Conclusions

Catalase is a crucial enzymatic antioxidant that efficiently decomposes hydrogen peroxide into water and oxygen, thereby reducing oxidative stress and preventing cellular and molecular disintegration. This enzyme plays a critical role in maintaining cellular redox balance. Its dysregulation is implicated in the etiology and progression of various diseases, including neurodegenerative disorders, cardiovascular diseases, cancer, aging, and rheumatoid arthritis. Genetic variations in the catalase gene can influence its activity and impact the progression of these diseases. The delicate balance between the generation of reactive oxygen species (ROS) and antioxidant defenses like catalase is particularly vital in conditions like cancer, where catalase can play a dual role in the survival of both standard and cancer cells. Catalase’s therapeutic potential is significant in preventing and managing several diseases, emphasizing the need to enhance its stability, specificity, and longevity for effective antioxidant therapy.

However, the therapeutic use of catalase faces challenges related to its stability, bioavailability, and delivery to target tissues. Innovative strategies such as utilizing nanocarrier systems and making chemical modifications have been explored to address these challenges. Combining catalase with other antioxidants in covalently conjugated formulations has shown synergistic benefits, offering promising avenues for enhancing therapeutic effects in antioxidant therapy. Furthermore, catalase has potential benefits in maintaining fertility and supporting healthy pregnancies, although more research is required to understand its full impact on reproductive health. Addressing potential immunogenic responses and ensuring specific delivery are crucial for improving its efficacy and safety profile in therapeutic applications.

Catalase’s specificity for hydrogen peroxide might limit its efficacy under conditions where other reactive oxygen species contribute to oxidative stress, requiring broader targeting strategies. The complex interplay of catalase with endogenous systems necessitates a comprehensive understanding to avoid unintended consequences in therapeutic contexts. Future research should focus on further optimizing catalase’s therapeutic efficacy through advancements in nanotechnology, gene therapy, and protein engineering. Additionally, exploring catalase as a diagnostic or prognostic biomarker and its neuroprotective attributes in neurodegenerative diseases presents exciting research opportunities. Overall, catalase holds significant promise as a therapeutic target for disease prevention and management, warranting continued investigation in this field.

## Figures and Tables

**Table 1:** Various studies show the modulation of catalase by different approaches.

Investigation	Approach	Outcome of the Study	Reference
Melanoma	Multifunctional immunoliposomes (CAT@aPDL1-SSLs)	Inhibition of the growth of tumors and increased survival of mice	[189]
Several neurodegenerative diseases and brain injury	Poly(lactic co-glycolic acid)	Inhibition of changes in the structure of the cell membrane, DNA damage, protein oxidation, mitochondrial membrane transition pore opening, and neurite network	[181]
Tumor hypoxia	Liposomes constituted by cisplatin(IV)-prodrug-conjugated phospholipid, forming CAT@Pt(IV)-liposome	Alleviation of hypoxia within the tumor for improved cancer chemotherapy and radiation	[201]
Biophysical, theoretical, and cellular studies of human mesenchymal cells and catalase	Interaction of SiO_2_ NPs with CAT and human MSCs (hMSCs)	SiO_2_ nanoparticles had very little effect on mortality, and they may be valuable carriers for catalase administration to hMSCs	[191]
Oxidant lung injury	SOD and catalase-containing liposomes	Delivery of enzymes can reduce tissue damage caused by ROS	[202]
Tumor microenvironment	Liposomal encapsulated catalase (CAT), lyso-targeted NIR photosensitizer (MBDP), and doxorubicin (DOX), forming FA-L@MD@CAT	Greatly accelerate tumor death, reverse the immunosuppressive tumor microenvironment, and improve cancer chemotherapy and photodynamic treatment	[194]
H1N1 influenza-induced pneumonia in mice	PEG-recombinant human catalase (PEG-rhCAT)	Compared to native rhCAT, PEG-recombinant human catalase (PEG-rhCAT) was more effectively administered and was linked to a greater survival ratio, fewer severe lung damage, lower ROS levels, and less viral multiplication.	[192]

**Table 2:** Various studies showing combination approaches for improving the antioxidant activities of catalase.

Investigation	Synergistic Molecule (s)	Observed Effects	Reference
Rat arterial thrombosis model	Superoxide dismutase-chondroitin sulphate and catalase-chondroitin sulphate covalent conjugates	Simple and effective protection of the vascular wall against various injuries with the use of the covalent conjugate superoxide dismutase-chondroitin sulphate-catalase	[204]
A549 human lung adenocarcinoma cells	Cisplatin, 5-fluorouracil, paclitaxel, daunorubicin, and hydroxyurea	Combining CAT (or CAT analogs) with traditional chemotherapeutic drugs, especially cisplatin, is a promising therapeutic strategy for the treatment of lung cancer	[205]
HepG2 cells	Doxorubicin (DOX)	High DOX transport efficacy, efficient CAT activity modulation, and increased cytotoxicity towards HepG2 cells	[61]
Tumor microenvironment	Doxorubicin (DOX)	Substantially accelerate tumor death, reverse the immunosuppressive environment within the tumor, and boost cancer chemo-photodynamic treatment	[195]
Liver nonparenchymal cells	Superoxide dismutase derivatives	Injecting mannosylated superoxide dismutase into the body slightly enhanced the catalase derivatives’ inhibitory effects.	[206]

## Data Availability

Data are contained within the article.
